# Supplementary material for: Splice-Junction-Based Mapping of Alternative Isoforms in the Human Proteome
Source: Cell Rep. Author manuscript; Available in PMC 2020 Jan 15. (PMC6961840; doi:10.1016/j.celrep.2019.11.026)
Supplement: 2 [file NIHMS1546469-supplement-2.zip › DF1/PXD009737/TestisGluC/TestisGluC_6_PPP4R1_RCFVSDRTISLQQATQYSE.pdf]

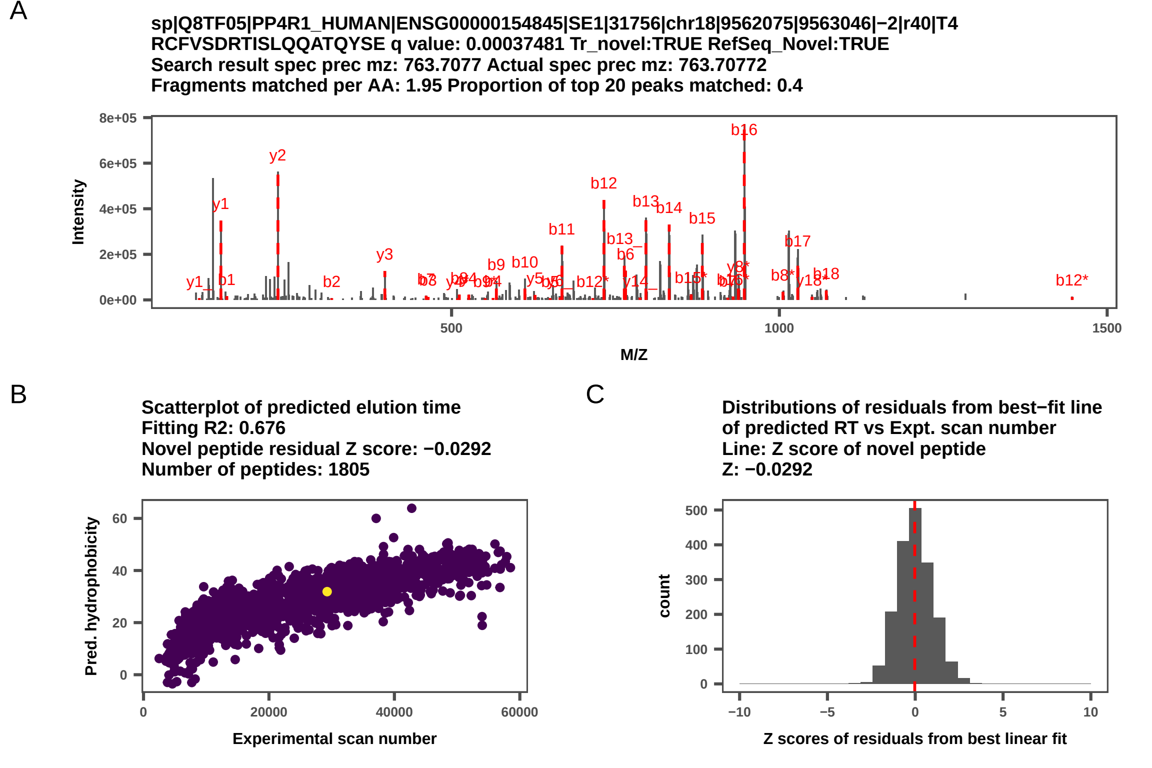
A
sp|Q8TF05|PP4R1_HUMAN|ENSG00000154845|SE1|31756|chr18|9562075|9563046|−2|r40|T4
RCFVSDRTISLQQATQYSE q value: 0.00037481 Tr_novel:TRUE RefSeq_Novel:TRUE
Search result spec prec mz: 763.7077 Actual spec prec mz: 763.70772
Fragments matched per AA: 1.95 Proportion of top 20 peaks matched: 0.4
Intensity
8e+05
6e+05
4e+05
2e+05
0e+00
M/Z
500
1000
1500
y1
y2
y3
b1
b2
b9
b10
b11
b12
b13
b14
b15
b16
b17
b18
b12*
B
Scatterplot of predicted elution time
Fitting R2: 0.676
Novel peptide residual Z score: −0.0292
Number of peptides: 1805
Pred. hydrophobicity
60
40
20
0
Experimental scan number
0
20000
40000
60000
C
Distributions of residuals from best−fit line
of predicted RT vs Expt. scan number
Line: Z score of novel peptide
Z: −0.0292
count
500
400
300
200
100
0
Z scores of residuals from best linear fit
−10
−5
0
5
10
